# CLGB-Net: fusion network for identifying local and global information of lesions in digital mammography images

**DOI:** 10.3389/fonc.2025.1600057

**Published:** 2025-07-17

**Authors:** Ningxuan Hu, Zhizhen Gao, Zongyu Xie, Lei Li

**Affiliations:** ^1^ School of Medical Imaging, Bengbu Medical University, Anhui, China; ^2^ Department of Radiology, The First Affiliated Hospital of Bengbu Medical University, Anhui, China

**Keywords:** breast cancer, early screening, CAD, deep learning, CLGB-Net

## Abstract

Worldwide, breast cancer ranks among the cancers with the highest incidence rate. Early diagnosis is crucial to improve the survival rate of patients. Digital Mammography (DM) is widely used for breast cancer diagnosis. The disadvantage is that DM relies too much on the doctor’s experience, which can easily lead to missed diagnosis and misdiagnosis. In order to address the shortcomings of traditional methods, a CLGB-Net deep learning model integrating local and global information is proposed for the early screening of breast cancer. Four network architectures are integrated into the CLGB-Net model: ResNet-50, Swin Transformer, Feature Pyramid Network (FPN), and Class Activation Mapping (CAM). ResNet-50 is used to extract local features. The Swin Transformer is utilized to capture global contextual information and extract global features. FPN achieves efficient fusion of multi-scale features. CAM generates a class activation weight matrix to weight the feature map, thereby enhancing the sensitivity and classification performance of the model to key regions. In breast cancer early screening, the CLGB-Net demonstrates the following performance metrics: a precision of 0.900, recall of 0.935, F1-score of 0.900, and final accuracy of 0.904. Experimental data from 3,552 samples, including normal, benign, and malignant cases, support these results. The precision of this model was improved by 0.182, 0.038, 0.023, and 0.021 compared to ResNet-50, ResNet-101, Vit Transformer, and Swin Transformer, respectively. The CLGB-Net model is capable of capturing both local and global information, particularly in terms of sensitivity to subtle details. It significantly improves the accuracy and robustness of identifying lesions in mammography images and reduces the risk of missed diagnosis and misdiagnosis.

## 1 Introduction

Breast cancer is one of the most common cancers in the world. According to the World Cancer Report 2020 issued by the International Agency for Research on Cancer (IARC), the prevalence of breast cancer exceeded that of lung cancer, ranking first in the world (1). By 2020, more than 2.3 million women worldwide have been diagnosed with breast cancer. According to the study, among the 2.3 million patients in the same year, 700000 cases died as a result (2). The Report of the breast cancer Committee of the Lancet estimates that by 2040, there will be more than 3 million new cases of breast cancer every year, and it is estimated that there will be more than 1 million new deaths (3). In view of the high incidence rate and mortality of breast cancer, early detection and diagnosis are particularly important. Breast cancer has no obvious symptoms in the early stage, but most patients are in the late stage when diagnosed, which leads to a high mortality rate. The American Cancer Society pointed out that the five-year relative survival rate of patients with early breast cancer is as high as 99%. Therefore, early detection, diagnosis and treatment of breast cancer are crucial to improve the survival rate of patients (4).

At present, the early detection of breast cancer mainly depends on imaging examinations, such as digital mammography (DM), digital breast tomography (DBT), magnetic resonance imaging (MRI), ultrasound (US), nuclear magnetic resonance technology and their combination technologies (2). Because of the high resolution, simple operation, repeatability and non-invasive characteristics of DM, DM is widely used in breast cancer screening. However, when using DM for breast cancer diagnosis, the results depend heavily on imaging physicians’ experience. This can affect diagnostic accuracy. It may even lead to unnecessary irradiation and invasive examinations for further diagnosis (5, 6). In order to address these challenge, computer-aided diagnosis (CAD) systems have gradually become a research hotspot (7, 8). In DM, CAD can assist radiologists in image interpretation and diagnosis with the help of computer algorithms, especially for marking and distinguishing benign and malignant lesions. CAD can not only improve the efficiency and accuracy of detection and diagnosis of breast cancer, but also play an important role in improving image quality, histological classification and predicting patient prognosis (2, 5). By applying artificial intelligence (AI) for DM image analysis, the application of CAD systems can avoid adverse reactions caused by additional irradiation in patients, and reduce unnecessary invasive examinations such as biopsies. In recent years, CAD has been gradually applied to the early screening of breast cancer (4, 9, 10). Among many methods, deep learning (DL) has been widely used in the early screening of breast cancer. Deep learning algorithms can automatically extract information from data, autonomously select the best features of images, and are quickly applied to DM based auxiliary diagnosis due to their characteristics (4). Deep learning technology has received extensive attention in the CAD system of medical image analysis. Significant research achievements have been made in image detection of various diseases, such as breast cancer detection (11, 12), neoadjuvant chemotherapy response and axillary lymph node metastasis prediction of breast cancer (13), colorectal polyp detection (14), skin cancer detection (15), brain tumor classification (16), etc.

There are still some shortcomings in the comprehensive analysis of DM image diagnosis. These shortcomings include high requirements for the experience level of radiologists, risks of misdiagnosis and missed diagnosis, and additional exposure and invasive examinations. In response to the above issues, this study proposes a new breast lesion recognition model: CLGB-Net deep learning network. This model efficiently integrates local and global features, comprehensively captures subtle information, and has the ability to accurately locate key areas, demonstrating significant advantages in clinical diagnosis of DM images. The advantage of the CLGB-Net deep learning network model is that it focuses on local ROI regions, increases the weight of effective information, and thus improves the accuracy of early screening. The proposal of this model not only overcomes the shortcomings of traditional methods that overly rely on doctors’ clinical experience, but also demonstrates advantages in early disease screening.

## 2 Related work

With the rapid development of medical imaging technology and its widespread application in clinical diagnosis, especially the introduction of computer technology and artificial intelligence, significant breakthroughs have been made in the field of medical image processing and analysis. In the field of image recognition, manual fine annotation of data images provides an important basis for the application of deep learning, especially in breast cancer detection and treatment planning. High quality manual annotation significantly improves the accuracy and reliability of the model, enabling it to learn disease features more accurately, thus playing a key role in early detection and precise treatment planning.

### 2.1 Recent research based on manually annotated data

Manual annotation not only enhances the learning ability of algorithms, but also provides valuable auxiliary tools for medical professionals. Sigrun et al. (17) used a DL segmentation model. The model was based on 3D CNN U-net. It automatically segmented target areas and risk organs (OARs). This was applied to the local area of breast cancer radiotherapy. This study used the CT image data of 200 patients with left breast cancer in two Norwegian hospitals. Three clinical oncologists and three radiation therapists manually delineated the geometric similarity indicators of seven clinical target areas (CTVs) and eleven organs at risk (OARs). The results showed that for most structures, the 3D CNN U-net model performed significantly better than inter observer variability (IOV). In clinical evaluation, 85% of CTVs and 98% of OARs do not require or only require minor modifications. In addition, the model significantly reduces manual sketching time from about 1 hour to 15 minutes. Therefore, this model can not only significantly improve work efficiency, but also maintain high-quality segmentation accuracy.

Besides, Aboutalib et al. (18) analyzed the mammographic images of two independent data sets, FFDM (Full Field Digital Mammography) and DDSM (Digital Database for Screening Mammography), using the deep convolution neural network (CNN) model based on the AlexNet structure in response to the problem of high false recall rate in breast cancer screening. 14860 images of 3715 patients in two independent datasets were initially manually annotated by experienced radiologists. In the FFDM dataset, the AUC range is 0.66-0.81. In the DDSM dataset, the AUC range is 0.77-0.90. When fusing FFDM and DDSM datasets, the AUC range is 0.76-0.91. In addition, when applying the incremental transfer learning strategy (pre-trained with ImageNet and fine-tuned with DDSM dataset) on the FFDM dataset for testing, the performance of malignant and recall benign models improved from 0.75 to 0.80. The results show that the deep convolution neural network (CNN) model based on the AlexNet structure can effectively identify subtle imaging features and reduce unnecessary recalls, thus improving the efficiency and accuracy of breast cancer screening. Nusrat (19) et al. used ensemble deep learning models (including DenseNet-121, DenseNet-169, ResNet-101v2, and ResNet-50) to detect and grade invasive ductal carcinoma (IDC). Pathologists manually mark the IDC (invasive ductal carcinoma) area in the full section image of breast cancer stained by H&E to determine which areas are positive or negative for IDC and their corresponding cancer grades. Through experiments on the Agios Pavlos and BreakHis datasets, the integrated model, DenseNet-121 and DenseNet-169 combined with test time augmentation (TTA), achieved an accuracy of 94.05%, an F1-score of 95.70%, and a balanced accuracy of 92.70% in IDC detection tasks. The accuracy of using this model improved by 1.58% and 2.62% compared to using DenseNet-121 and DenseNet-169 separately. In the IDC grading task, the integrated models (including DenseNet-121, DenseNet-201, ResNet-101v2, and ResNet-50) achieved the highest accuracy of 69.31% to 79.14% at different magnifications on the Databiox dataset, and achieved an overall accuracy of 89.26% on the Agios Pavlos dataset, far higher than the results of other benchmark models. These data fully demonstrate the significant advantages and robustness of the integrated model in improving detection and grading accuracy.

The above research results indicate that the advantage of manual annotation lies in providing a high-quality and accurate data foundation, which is crucial for training deep learning models. High quality manual annotation significantly improves the accuracy and reliability of the model, enabling it to learn disease features more accurately, thus playing a key role in early detection and precise treatment planning. However, in practical applications, manual annotation has drawbacks such as long process time, significant manpower investment, and difficulty in ensuring consistency, and highly relies on the clinical experience of experts. This dependency not only increases costs, but may also result in uneven labeling quality due to human factors. To overcome the limitations of manual annotation, researchers have begun exploring a new method for automated annotation that does not rely on manual labor.

### 2.2 Recent research based on automatic feature search

The rapid development of methods such as artificial intelligence CAD has promoted its application in the field of medical imaging. Experts and scholars are increasingly inclined to explore a method that combines deep learning and automation technology to compensate for the shortcomings of manual labor. Related research attempts to gradually use advanced algorithms and model structures, such as supervised learning, transfer learning and other methods to reduce the dependence on manual annotation, and improve the processing ability and adaptability of the model in complex scenes. As early as 2021, William (20) and others took the lead in the research based on the weakly supervised learning framework. The OMI-DB dataset and DDSM dataset were used in this study. Image annotation is mainly realized by weak supervised learning framework, which reduces the dependence on traditional manual fine annotation. Researchers use a small number of expert labeled data to generate the initial model, and use this model to generate high-quality pseudo tags for large-scale unlabeled data. On OMI-DB dataset, the AUC value and sensitivity of this method are 0.94 and 89%, while on DDSM dataset, the AUC value is 0.92 and the sensitivity is 87%. Compared with the traditional manual annotation method, the AUC value of this method is increased by 0.03, and the sensitivity is increased by 4%. Üzen (21) et al. Used several public breast tumor image datasets, including Mini-MIAS (MIAS), Curated Breast Imaging Subset of Digital Database for Screening Mammography(CBIS-DDSM) and Breast Ultrasound Tumor Dataset (BreaST). Image processing and classification mainly rely on automated methods, but still use some manually labeled data as the basis to ensure the initial training quality and accuracy of the model. Experimental results show that the model achieves 97.5% accuracy and 97.0% F1-score on public data sets (such as MIAS, CBIS-DDSM and BreaST). Compared with ResNet-50 and U-Net, the accuracy of the model was improved by 3.7% and 4.9% respectively, and the F1-score was improved by 3.8% and 5.2% respectively. This research not only improves the performance of the model, but also reduces the dependence on a large number of manual annotation data. Aiming at the problems of breast density estimation and breast cancer risk assessment, Omid (22) and others used the Deep-LIBRA method to analyze the multi-ethnic and multi-agency data sets containing 15661 FFDM images (from 4437 women). In this study, deep learning combined with radiomics feature analysis was used for automatic image processing, including background removal, pectoralis major muscle removal, and dense and non-dense tissue segmentation. This method uses deep learning technology, especially U-Net convolutional neural network architecture, image processing and machine learning technology based on radiomics to achieve background removal, pectoralis major muscle removal, and segmentation of dense and non-dense tissue regions. The results showed that on the independent case-control data set, the estimated breast density percentage (PD) generated by deep Libra was highly correlated with the results of expert film reading (Spearman correlation coefficient=0.90). Moreover, in the breast cancer risk assessment model adjusted for age and BMI, the AUC of deep Libra reached 0.612, which was significantly better than the other four commonly used commercial and open source breast density assessment methods (AUC range was 0.528 to 0.599). These results clearly show that this method not only effectively reduces the dependence on massive manual annotation data, but also significantly improves the detection accuracy and cross dataset generalization performance by optimizing the model architecture and training strategy and improving the model performance, which fully demonstrates its technical advancement and practical value in the automatic breast cancer detection task, and provides a more efficient and reliable solution for medical image analysis.

In response to the shortcomings of the aforementioned research, this study proposes a deep learning method based on the deep learning method of automatic label generation. This method specifically combines advanced technologies such as ResNet-50, Swin Transformer, FPN and CAM like to form a new breast disease diagnosis system called CLGB-Net. Through multi-scale feature extraction and global context information fusion, CLGB-Net can not only capture local detail features, but also effectively integrate global semantic information, which significantly improves the classification accuracy and robustness of breast lesion images. This method provides a more efficient and accurate solution for DM image analysis, while reducing the demand for computing resources and processing time. The model automatically generates high-quality labels through the iterative training process, replacing the traditional manual fine annotation. In each iteration, the model will automatically adjust its parameters to optimize the understanding and classification ability of the input data, and generate accurate and consistent labels. Unlike relying on experts for tedious and time-consuming manual annotation, this automated method not only greatly improves work efficiency and reduces the possibility of human error, but also can handle large-scale data sets. In addition, since there is no need for human intervention, this method saves researchers a lot of time and resources, so that they can focus on the optimization and improvement of the model. Therefore, the model significantly improves the efficiency and accuracy of DM image analysis through automatic label generation and advanced feature extraction technology. It not only provides researchers with powerful tools, but also brings new possibilities for clinical practice, and helps to promote the development of medical image analysis to a higher level.

## 3 Materials and methods

### 3.1 Data collection

Data collection was conducted in the Radiology Department of the First Affiliated Hospital of a medical university in Anhui Province. The data acquisition equipment is the Siemens MAMMAMMAT Inspiration mammography machine from Germany. The specific imaging parameters are as follows: tube current time is 92.5 mAs, pressure during breast compression is 67 N, thickness of imaging after breast compression is 44 mm, target/filter combination of tube exposure is W/Rh (tungsten/rhodium), incident dose is 3.7 mGy, and glandular dose is 1.27 mGy.

This study collected 1868 cases of breast mammography performed in the radiology department of hospitals from January 2019 to January 2024, all of whom were female. The study was approved by the Ethics Committee of Bengbu Medical University ([2024] No. 370). All images were anonymized by removing patient identifiers (such as name, ID number) and converting DICOM files to JPG format using MicroDicom v3.4.7. No personal health information was retained during this process. Among them, the age range of normal cases is 48.43 ± 9.10, the age range of benign cases is 48.18 ± 9.69, and the age range of malignant cases is 55.68 ± 10.54. A total of 3598 images were collected, including single and bilateral CC+MLO images of the same patient. 46 that did not meet the requirements were excluded, and 3552 images were finally obtained for analysis. All images were divided into three categories: normal, benign, and malignant. Among them, the control group had 503 normal cases and 1210 images, including 802 unilateral CC+MLO images and 408 bilateral CC+MLO images. There are a total of 547 benign cases with 1014 images, including 80 unilateral CC or MLO images of 80 cases and 934 unilateral CC+MLO images of 467 cases. There were 818 malignant cases with a total of 1328 images, including 308 unilateral CC or MLO images of 308 cases and 1020 unilateral CC+MLO images of 510 cases.

The collected data includes lesions confirmed by two clinical doctors with more than 10 years of diagnostic experience, classified as BIRADS grade 3 or below and without malignant tendency according to the 2013 version of BIRADS (23) guidelines, as well as lesions classified as BIRADS grade 4 or above. The benign and malignant classification of all images is based on pathological reports as the gold standard. The exclusion criteria are that the DM image quality does not meet the standard (such as blurry images, insufficient resolution) or the shooting range does not fully cover the lesion area, in order to ensure the high quality of the data and the reliability of the research results.

### 3.2 Image preprocessing and data augmentation

One of the most critical steps before conducting data analysis is data preprocessing and data augmentation, which can effectively improve model performance and ensure its generalization ability. Common data augmentation methods include rotation, scaling, horizontal flipping, vertical flipping, transposition, and cropping (7, 11). These operations not only help adjust image size to match model input requirements, but also simulate more diverse situations by introducing changes, thereby increasing the diversity of training samples.

There were 3552 mammogram images collected in this study, including training set 2842, test set 355, and validation set 355. For the training set, data augmentation techniques such as image scaling, random cropping and random flipping were adopted on the basis of image preprocessing. The validation and test sets are only preprocessed (scaled, normalized) on a basic basis to ensure that the test data is not exposed in an enhanced form during training. Data augmentation is dynamically applied in real-time during the training process (different augmentation samples are generated for each training cycle), while the validation/test set uses a fixed preprocessing process and is implemented through code isolation to ensure the fairness of model evaluation. Image scaling can adjust the image size to meet the input requirements of the model, while simulating targets of different scales. Random cropping increases sample diversity by cropping different parts of the image, enhancing the model’s ability to learn local features. Random flipping (including horizontal and vertical flipping) generates mirrored images, further enriching the training data and improving the model’s generalization performance. Normalization operation unifies the range of image pixel values, accelerates the model training process, and improves numerical stability. The comprehensive application of these preprocessing methods not only significantly improves the diversity and quality of data, enhances the recognition and classification performance of the model, but also ensures that the model further strengthens its ability to capture local features while extracting global information. The multi-scale and multi perspective data processing strategy enables the model to more accurately identify and analyze local details while maintaining understanding of the overall image, ultimately achieving better prediction performance and higher stability.

Finally, a batch data loader is built using the DataLoader module: the training set adopts the random disruption (shuffle=true) strategy to enhance the generalization ability of the model to the data distribution, while the validation set and the test set adopt the fixed sequence loading (shuffle=false) to ensure the certainty of the evaluation process and the reproducibility of the results. This design realizes the efficient management of data flow through dynamic batch processing mechanism (combined with multi-threaded acceleration and memory optimization), and finally forms a complete training verification test pipeline including data enhancement, batch standardization and hierarchical verification, which provides a systematic experimental framework for model development.

### 3.3 Construction of CLGB-Net model

The CLGB-Net model is a lesion diagnosis system for breast DM images that integrates local and global information. It is composed of ResNet-50, Swin Transformer, Feature Pyramid Network (FPN) and Class Activation Mapping (CAM). The following will explain each module separately:

#### 3.3.1 ResNet-50

ResNet stands for Residual Network. Increasing the network size can lead to gradient vanishing, while ResNet introduces Residual Blocks (7), which directly pass the input of the network to the output through Skip Connection (24), thereby solving the problems of gradient vanishing and gradient explosion in deep networks and achieving faster system training. ResNet comes in various forms, including ResNet-18, ResNet-34, ResNet-50, ResNet-101, and ResNet-152. ResNet-50 has 50 convolutional layers, and its deep structure enables it to learn complex features from data, thereby achieving high-precision image classification and object detection. It is a deep CNN with powerful feature extraction and classification capabilities.

**Figure 1:**
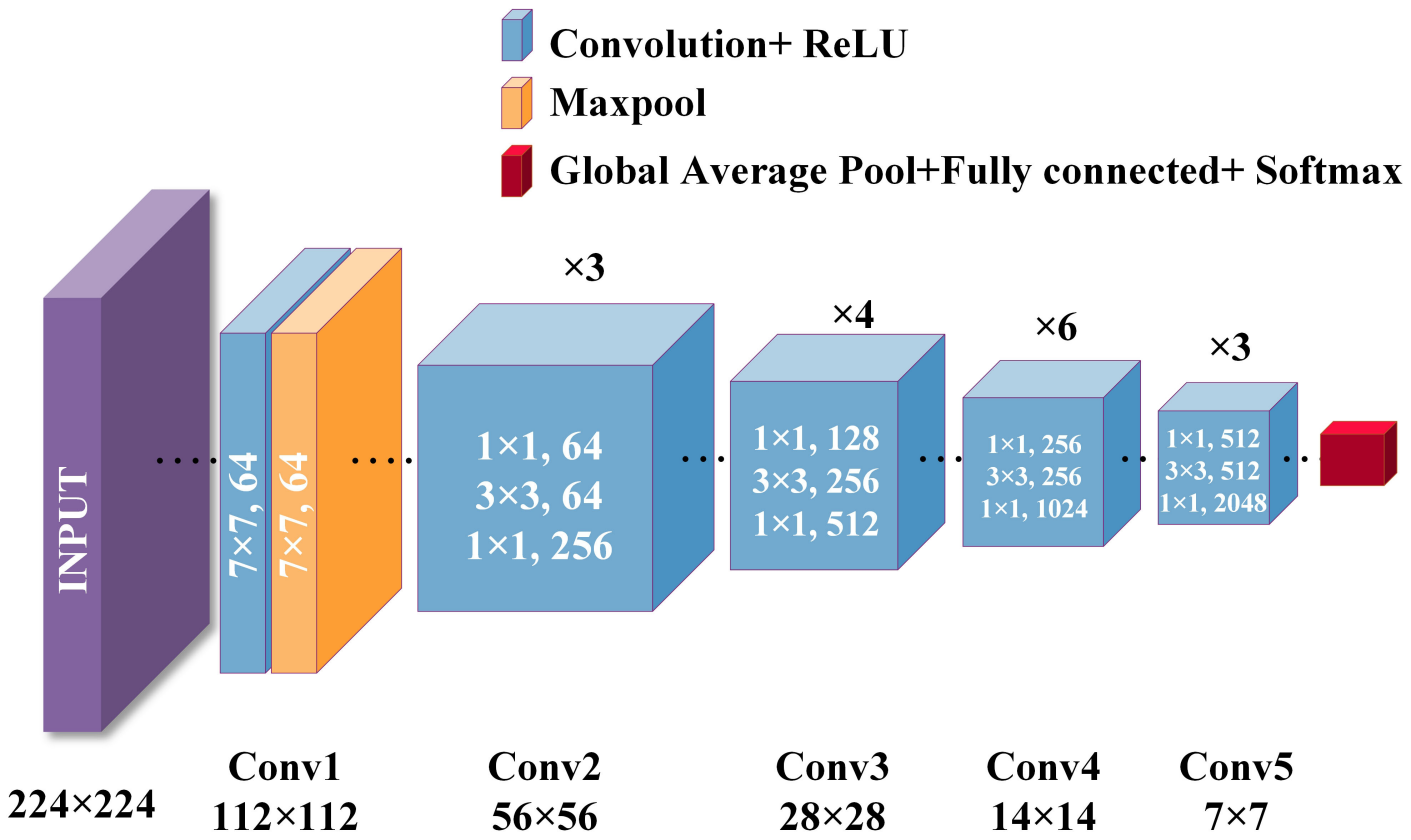
ResNet-50 structure diagram.

Considering the size of the dataset in this study, we chose to use the ResNet-50 architecture. ResNet-50 is a deep residual network consisting of 50 layers, mainly including an initial layer, residual block layer, global average pooling layer, and fully connected layer. The specific structure is shown in **Figure 1**.

x is the input image, $W_{conv1}$ is the weight of the convolutional layer, $b_{conv1}$ is the bias term of the convolutional layer, $x_{1}$ is the output after convolution and ReLU activation, and $x_{2}$ is the output after max pooling. The initial convolutional layer is shown in Equations 1, 2:


(1) $$x_{1}=ReLU(W_{conv1}\cdot x+b_{conv1})$$

(2) $$x_{2}=MaxPool(x_{1})$$

$x_{2}$ Representing the output of the max pooling layer, $W_{i}$ representing the weight matrix of the convolutional layer in the residual block; $F(x_{i},W_{i})$ representing the forward propagation function in the residual block, typically consisting of multiple convolutional layers and activation functions; $ReLU(y_{i})$ apply an ReLU activation function to the output of the $y_{i}$ residual block to make it nonlinear; ResBlock represents a residual block composed of a series of convolutional layers. The residual block layer is shown in Equation 3:


(3) $$x_{3}=ResBlock(x_{2},W_{r})$$

$x_{3}$ representing the output of the last convolutional layer or residual block; $x_{pool}$ represents the output after global average pooling. The global average pooling layer is shown in Equation 4:


(4) $$x_{pool}=GlobalAvgPool(x_{3})$$

$x_{pool}$ represents the output after global average pooling, $W_{k}$ represents the weight of the fully connected layer, $b_{k}$ represents the bias term of the fully connected layer, $y_{k}$ represents the output of the fully connected layer. The fully connected layer is shown in Equation 5:


(5) $$y_{k}=W_{k}\cdot x_{pool}+b_{k}$$

Therefore, the ResNet-50 structure is as shown in Equation 6:


(6) $$y_{k}=W_{k}\cdot GlobalAvgPool(ResBlock(MaxPool(ReLU(W_{conv1}\cdot x+b_{conv1}))))+b_{k}$$

The advantage of ResNet-50 is that it effectively solves the common problems of gradient vanishing and exploding in deep networks by introducing residual blocks and skipping connections, thereby achieving a faster and more stable training process. Its deep structure enables it to learn complex features, making it suitable for high-precision image classification and object detection tasks. ResNet-50 not only improves the performance of the model on large-scale datasets, but also enhances its stability and training efficiency, making it a powerful feature extraction and classification tool. Therefore, this study utilized ResNet-50 to efficiently process breast lesion images, significantly improving classification accuracy and model generalization ability.

#### 3.3.2 Swin Transformer

**Figure 2:**
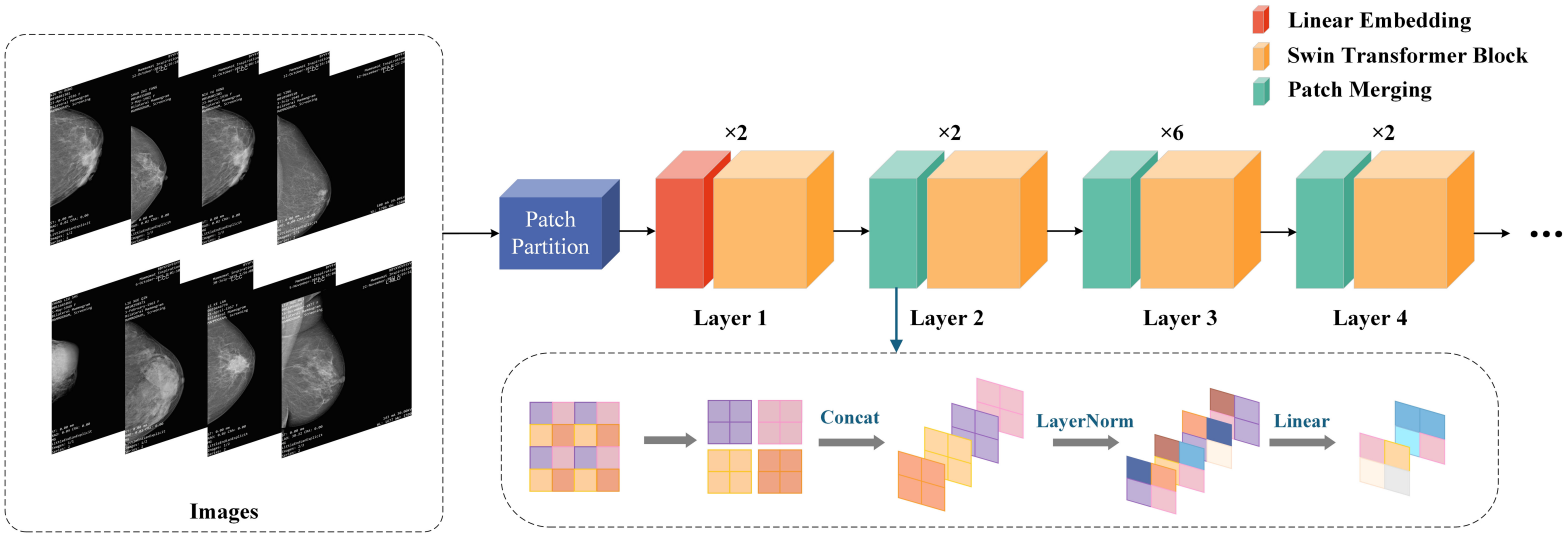
Swin Transformer structure diagram.

In the field of computer vision, although CNNs have long been the dominant architecture, in recent years, Transformer networks have shown significant advantages in computer vision and natural language processing (NLP) by utilizing self-attention mechanisms to extract long-range dependencies (11), more and more research is exploring the possibility of applying Transformers to visual tasks. Compared to traditional CNNs, Swin Transformer introduces the concept of “window” and combines “local attention” and “global attention” to capture more local and global feature (25). The uniqueness of Swin Transformer lies in its outstanding performance in learning attention based hierarchical features. As a hierarchical Transformer, Swin Transformer can serve as the foundational architecture for various computer vision tasks through mobile window computation. Its basic operations include window interaction, window interaction update, local window attention, and global window attention, establishing associations between windows through application window interaction (26). Given the ability of Swin Transformer to extract hierarchical multi-scale attention features, it is able to demonstrate top-level performance in complex computer vision tasks (14, 27). Its biggest advantage lies in the ability to extract multi-level and multi-scale attention features (20), which can capture more global information. This not only enhances the model’s ability to understand complex scenes, but also significantly improves its performance in various visual tasks such as image classification and object detection. The specific structure of Swin Transformer is shown in **Figure 2**.

CLGB-Net first divides the image into multiple patches in the Patch Partition module, then flattens the image in the channel direction, performs linear transformation on the channel data of each pixel through the Linear Partition layer, and then constructs feature maps of different sizes through Layers 1-4. Among them, only Layer 1 passes through a Linear Partition layer first, while Layers 2–4 are down sampled through the Patch Partition layer first. Finally, stack Swin Transformer Blocks repeatedly to obtain the final output.

Patch Partition is a non-convolutional down sampling technique used in Swin Transformer. As mentioned earlier, in Layers 2-4, a Patch Partition layer is first passed through. As shown in the figure below, if a 4x4 image is input into Patch Merging, the image is divided into 4 2x2 patches, and the pixels of the same color in each patch are combined together to obtain 4 feature maps. Concat and concatenate these four feature maps, pass them through a LayerNorm layer, and then perform linear changes in the depth direction of the feature map through a fully connected layer. Therefore, after passing through Patch Partition, the width and height of the image are reduced to half of their original size, while the depth is doubled.

In summary, Swin Transformer, by introducing a unique window shift mechanism, greatly enhances the model’s ability to capture long-range dependencies and multi-level information in images while maintaining efficiency, making it one of the important research directions in the current field of computer vision. This method provides a more flexible and powerful tool for future visual tasks.

#### 3.3.3 FPN

**Figure 3:**
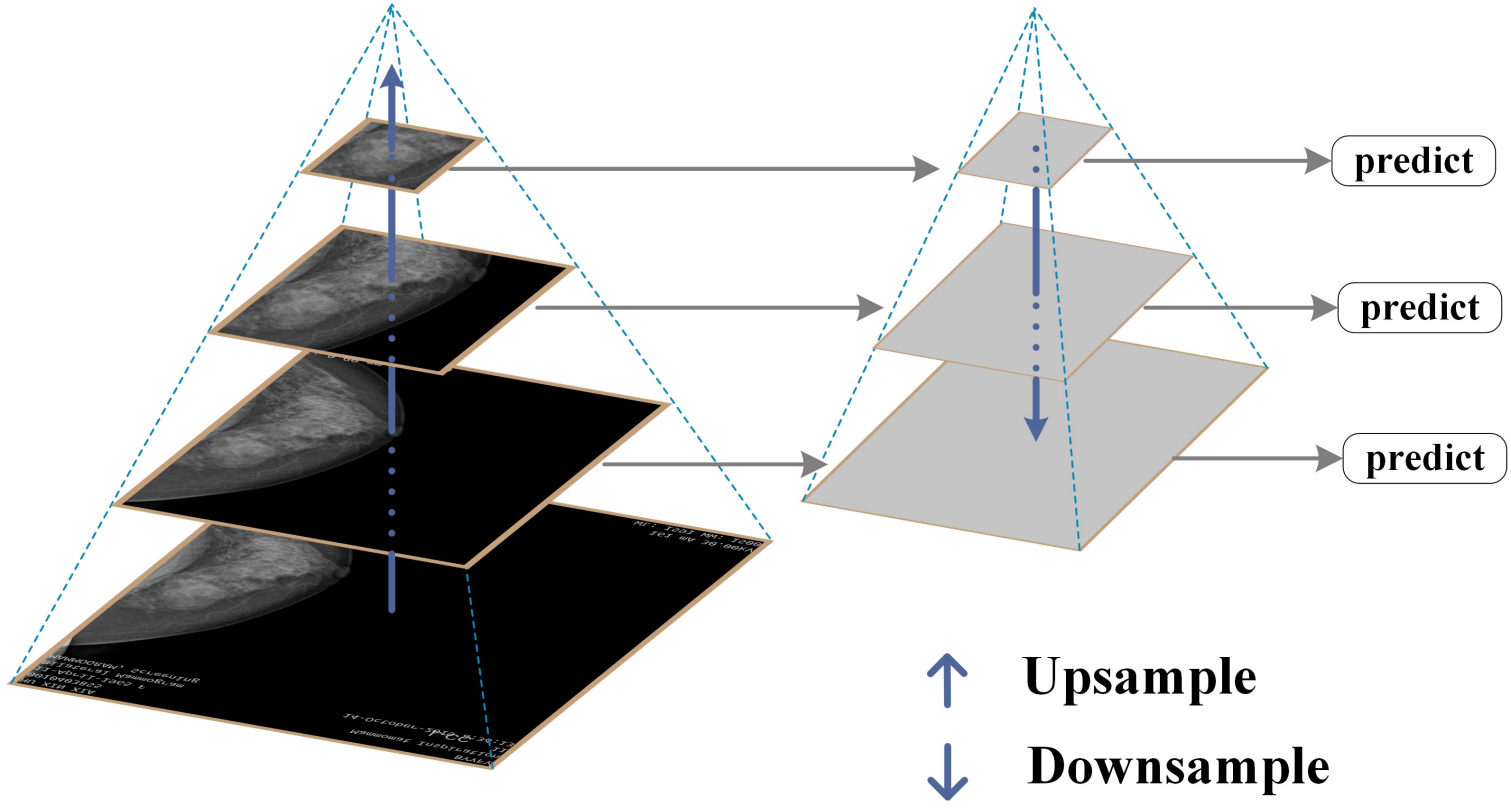
FPN structure diagram.

FPN is a fundamental component used in recognition systems to detect objects of different scales. It is one of the representative detectors in recent years and is a multi-scale feature representation method. Its biggest feature is its top-down feature fusion path and multi-scale detection paradigm (27). It has been proven to be very effective in extracting multi-scale features (28), and has played an important role in fields such as object detection, instance segmentation, and semantic segmentation (26). With its unique top-down feature fusion mechanism and multi-scale detection framework, FPN has become an indispensable component of modern detection systems and one of the most representative architectures in the field of object detection (29). The specific structure of FPN is shown in **Figure 3**.

This study constructs an image pyramid by generating images of different sizes after inputting the original image. Features are extracted from each layer of the image pyramid, and different sizes of features are extracted for prediction. The prediction results for all sizes are then statistically analyzed. Finally, several features are fused and several images are upsampled to obtain features of the same size. These features are then added together to obtain the output of the feature pyramid, which has the same size as the first layer. This feature enables FPN to better recognize small targets.

FPN effectively integrates deep high semantic information with shallow high resolution information through a top-down path and horizontal connection mechanism, generating multi-scale feature maps and significantly enhancing the model’s detection ability for targets of different sizes. It is particularly suitable for identifying small lesion areas in breast lesion images. In this study, by combining FPN with ResNet-50, the aim is to further improve the model’s ability to extract multi-scale image features, solve the problems of multi-scale lesion detection and small object recognition, enhance the stability and generalization ability of the model, and thus improve the accuracy and reliability of breast lesion classification.

#### 3.3.4 CAM

**Figure 4:**
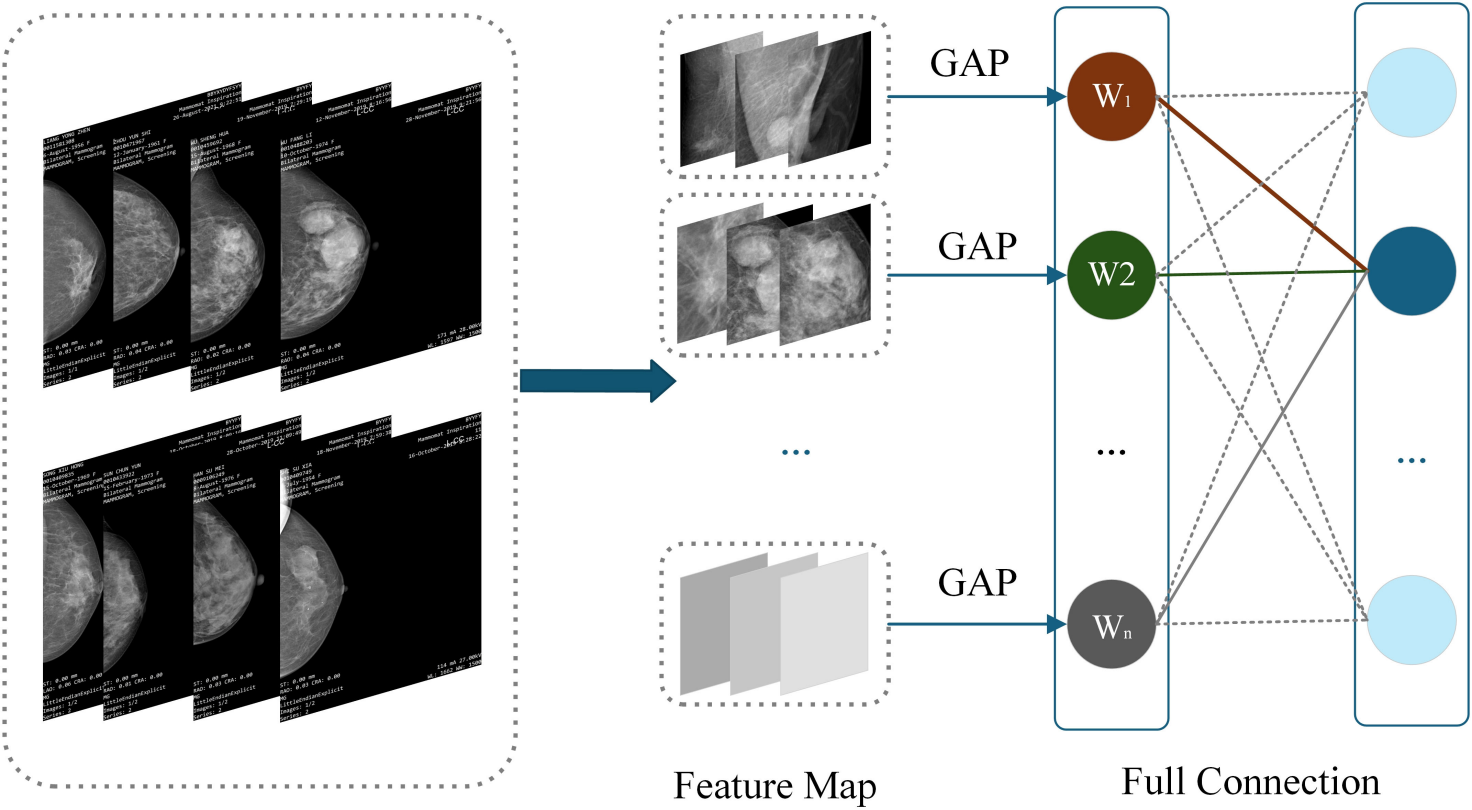
CAM structure diagram.

CAM is a technique that utilizes the output results of a model to activate important features in specific regions of the original image (30), It generates results by weighting and summing the last activated image of each category in the network using the weights of fully connected layers. CAM can generate heat maps that can locate the approximate location of lesion areas (31), demonstrating significant effectiveness, especially in image label based target localization work (29). CAM can identify and highlight discriminative regions in the input image, and predict category scores through visualization, emphasizing the key regions detected by CNN. This method enables CNNs trained for classification to learn and perform object localization without the need for bounding box annotation (32). The specific structure of CAM is shown in **Figure 4**.

The heatmap generated by CAM is a visual representation of the same size as the original image, with each pixel having a value range between 0 and 1, and is typically displayed through a grayscale map (0 to 255 gray levels). This heatmap is generated by weighted summation of feature maps extracted from the ResNet-50 network to determine which regions contribute significantly to the final classification task. Specifically, CAM utilizes the weights of fully connected layers to weight and sum the feature maps of the last convolutional layer, thereby generating a heatmap to highlight key regions in the input image. The identification of these key regions helps the model gradually optimize during the training process, enabling it to focus more accurately on local features that are crucial for classification tasks. Through this approach, CAM not only enhances the interpretability of the model, but also improves its performance for object detection tasks. It enables CNN trained through classification to learn and perform object localization without the need for bounding box annotation. This method enables CNN to effectively identify and locate discriminative regions in images, thereby improving overall classification accuracy and reliability. Therefore, CAM has demonstrated its powerful application potential in multiple fields such as medical image analysis and object recognition.

W_1_ to W_n_ represent the weight of the target category (breast lesions in this study) between each feature map of the last convolutional layer and the classifier. Since the feature vector output by GAP comes directly from the feature map, this weight can be regarded as the contribution of the feature map to the target category score. Weighted summation can be used to obtain CAM. $f_{k}(x,y)$ is the value of the (x,y) location of the last convolutional feature map, $w_{k}^{c}$ representing the k weights corresponding to the c class in the fully connected output layer. CAM formula such as Equation 7:


(7) $$P_{c}(x,y)=\sum kw_{k}^{c}f_{k}(x,y)$$

Due to its simplicity and intuitive visualization effect, CAM performs well in interpreting localization and classification tasks. CAM based algorithms have been widely applied in various studies due to their simplicity and intuitive visualization characteristics, in order to learn and recognize useful feature information in datasets more effectively and perform classification tasks (33). In summary, CAM not only simplifies the model training process, but also enhances the understanding of model behavior, making it an indispensable part of modern computer vision research. As a simple yet powerful tool, it provides higher interpretability and practicality for deep learning models, promoting their widespread application in multiple fields. In this study, after continuous training and updating of feature weights, the model can be created into a heatmap by CAM to demonstrate the effectiveness of the prediction results.

#### 3.3.5 CLGB-Net construction

**Figure 5:**
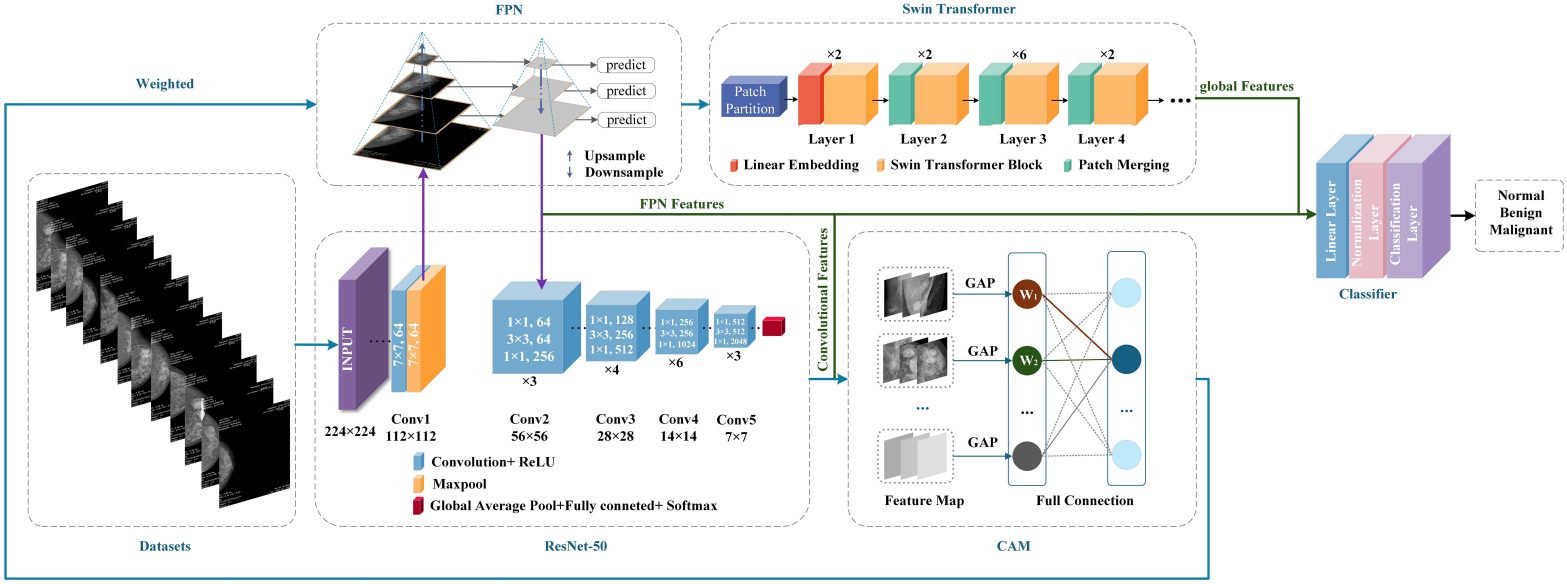
CLGB-Net technology roadmap.

CLGB-Net is based on the PyTorch framework and fully utilizes the deep feature extraction capability of ResNet-50, the global context modeling capability of Swin Transformer, the multi-scale feature fusion capability of FPN, and the interpretability of CAM to achieve accurate diagnosis of breast lesion images. **Figure 5** illustrates the technical circuit diagram of CLGB-Net.

The training process of CLGB-Net is divided into three sequential stages. The first stage is local feature extraction and CAM weight generation. Input processing: Mammography images are fed into ResNet-50 to extract hierarchical local features. CAM Activation: A CAM weight matrix is generated from the last convolutional layer of ResNet-50 to highlight areas of the image that are critical for classification. The second stage is FPN-based multi-scale feature fusion. The local features extracted by ResNet-50 will generate a multi-scale feature map through the FPN. These feature maps encode lesion details at different resolutions, enabling simultaneous detection of small lesions and large lesion areas. The third stage is global-local feature alignment and optimization. In order to solve the problem of alignment between FPN multi-scale features and Transformer feature maps, three optimization methods were constructed for CLGB-Net.

The first method is multi-scale resolution matching, which performs bilinear interpolation on the pyramid features output by FPN to achieve the same resolution as the high-level features of Swin Transformer, with the aim of eliminating scale differences. The second method is attention screening and dynamic fusion, using channel attention module (SE Block) to screen key scale information, dynamically allocating fusion weights of FPN and Transformer features through learnable parameters, and optimizing feature contributions at different lesion scales. The third method is the degree balancing strategy, which applies weak gradient weights to the shallow high-resolution features of FPN, suppresses local noise interference, and enhances the learning of deep semantic features. The above strategy effectively solves the representation differences and computational conflicts between multimodal architectures through feature adaptation, dynamic fusion, and computational optimization, achieving efficient collaborative modeling of local-global features.

Secondly, after global average pooling, the features are weighted with the weights calculated by CAM, and upsampling is used to make the feature maps consistent in size. Then, the weighted feature map is convolved by 1 × 1 to adjust the number of channels, and input into Swin Transformer to extract global contextual information. In order to better integrate the sequence feature processing of ResNet-50 with Swin Transformer’s block based self-attention mechanism in the CLGB-Net model, feature dimension alignment, block-based feature recombination, and cross modal attention guidance have been added for processing. Among them, the number of channels output by ResNet-50 is adjusted through a 1 × 1 convolutional layer to align with the embedding dimension of Swin Transformer, achieving dimension alignment. Subsequently, the 2D feature map of ResNet-50 was divided into non overlapping windows and transformed into serialized block features through linear projection as input to Swin Transformer, achieving a smooth connection between convolutional features and self-attention mechanism. Finally, residual connections are introduced in Swin Transformer to preserve local details, and cross attention mechanism is utilized to dynamically fuse global context and local features, enhancing their collaborative expression ability. Finally, the extracted local features, pyramid features, and global features are concatenated together to form a comprehensive feature vector for classifying benign, malignant, and normal lesions.

This study proposes a CLGB-Net architecture based on multi model fusion for the task of identifying breast lesions. A systematic strategy is adopted to address the issue of overfitting that may occur in the model. Firstly, at the model design level, CLGB-Net integrates ResNet-50, Swin Transformer, FPN, and CAM modules to construct a joint framework for multi-scale feature extraction and interpretability modeling. FPN achieves progressive fusion of cross level features, while CAM highlights key areas by generating heat maps, and the two work together to reduce redundant parameters and enhance the ability to distinguish subtle lesions. Secondly, at the training strategy level, the Cross Entropy Loss function is used to quantify the difference between the predicted and true labels, and combined with the stochastic gradient descent (SGD) optimizer for parameter updates. Hyperparameters refer to the parameters that need to be set before model training, which can control the process and structure of model training. Choosing the optimal hyperparameters plays an important role in improving the segmentation and classification efficiency of the model.

In order to achieve efficient collaborative modeling of local and global features and improve model generalization ability, this study sets the initial learning rate of the model to 0.001, ensuring the stability of parameter updates through a low initial learning rate, while using a periodic restart mechanism to avoid local minima. And dynamically adjust the learning rate through a cosine annealing learning rate scheduler to balance convergence speed and stability. The batch size is set to 64, which follows the theoretical framework of Mini batch stochastic gradient descent (Mini-batch SGD) and optimizes by balancing gradient noise suppression and memory utilization efficiency. Larger batches can reduce parameter update variance to stabilize convergence trajectories and avoid hardware memory overload caused by extreme batches. In addition, by combining the cross-entropy loss function with weight decay (L2 regularization), the parameter space is constrained to be complex. Finally, the necessity of each module was verified through ablation experiments, and the results showed that the fusion strategy significantly improved the classification accuracy and generalization performance of the model. The entire training process is implemented based on the PyTorch framework, ensuring efficiency and scalability. Through comparison, it was found that the CLGB-Net model, due to the introduction of Swin Transformer and FPN, compensates for the shortcomings of ResNet-50 in global context modeling and multi-scale feature fusion, and improves the model’s ability to capture complex lesion features. CLGB-Net combines ResNet-50’s local feature extraction with FPN’s multi-scale feature fusion, enhancing the model’s sensitivity to detailed information. By integrating ResNet-50 and Swin Transformer, the collaborative expression of local and global features has been further optimized. In summary, the CLGB-Net proposed in this study not only retains the interpretability guided by CAM heat maps, but also significantly improves classification accuracy and robustness through multi module fusion, making it more practical in the diagnosis of breast lesions.

## 4 Results and analysis

### 4.1 Evaluating indicator

**Figure 6:**
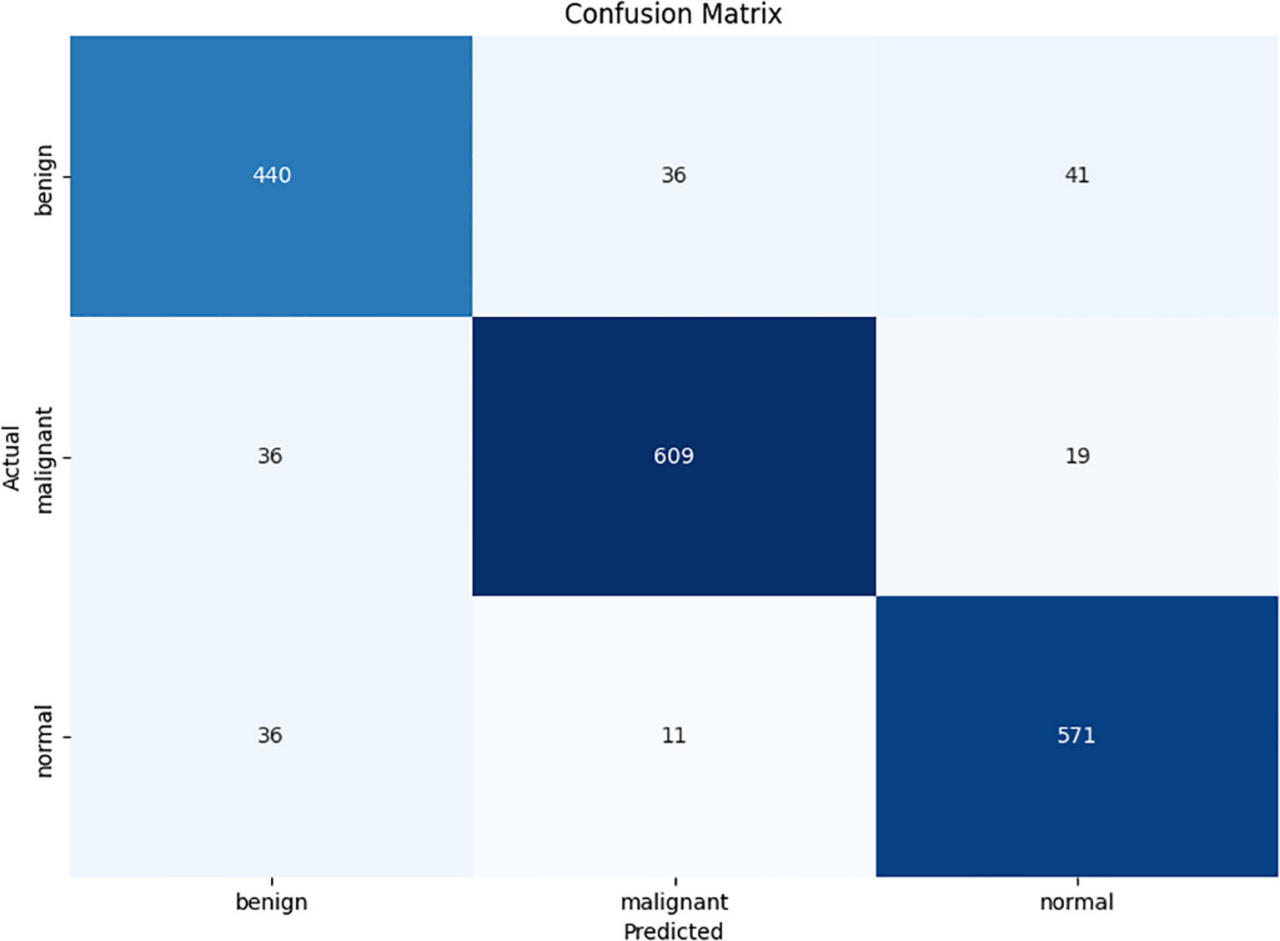
Confusion Matrix.

This experiment studied the confusion matrix of three categories and four performance indicators for each category to evaluate the performance of CLGB-Net, including accuracy, precision, recall, and F1-score. True Positive (TP) represents the number of samples correctly predicted as positive by the model, True Negative (TN) represents the number of samples correctly predicted as negative by the model, False Positive (FP) refers to the number of samples incorrectly predicted as positive by the model, and False Negative (FN) represents the number of samples incorrectly predicted as negative by the model. This study used the confusion matrix metrics in **Figure 6**. Overall, although the model can accurately distinguish these three categories in most cases, there is still room for improvement to reduce misjudgment rates and improve the overall recognition accuracy of the model.

t is the predicted category, and the performance indicators of Accuracy, Precision, Recall, and F1-score are defined in the Equations 8–11:


(8) $$Acc_{t}\text{= (}TP_{t}\text{+ }TN_{t}\text{) / (}TP_{t}\text{+ }FP_{t}\text{+ }TN_{t}+FN_{t})$$

(9) $$Pre_{t}=TP_{t}/(TP_{t}+FP_{t})$$

(10) $$Rec_{t}=TP_{t}/(TP_{t}+FN_{t})$$

(11) $$F_{1}-score_{t}=2\times (Pre_{t}+Rec_{t})/(Pre_{t}\times Rec_{t})$$

**Figure 7:**
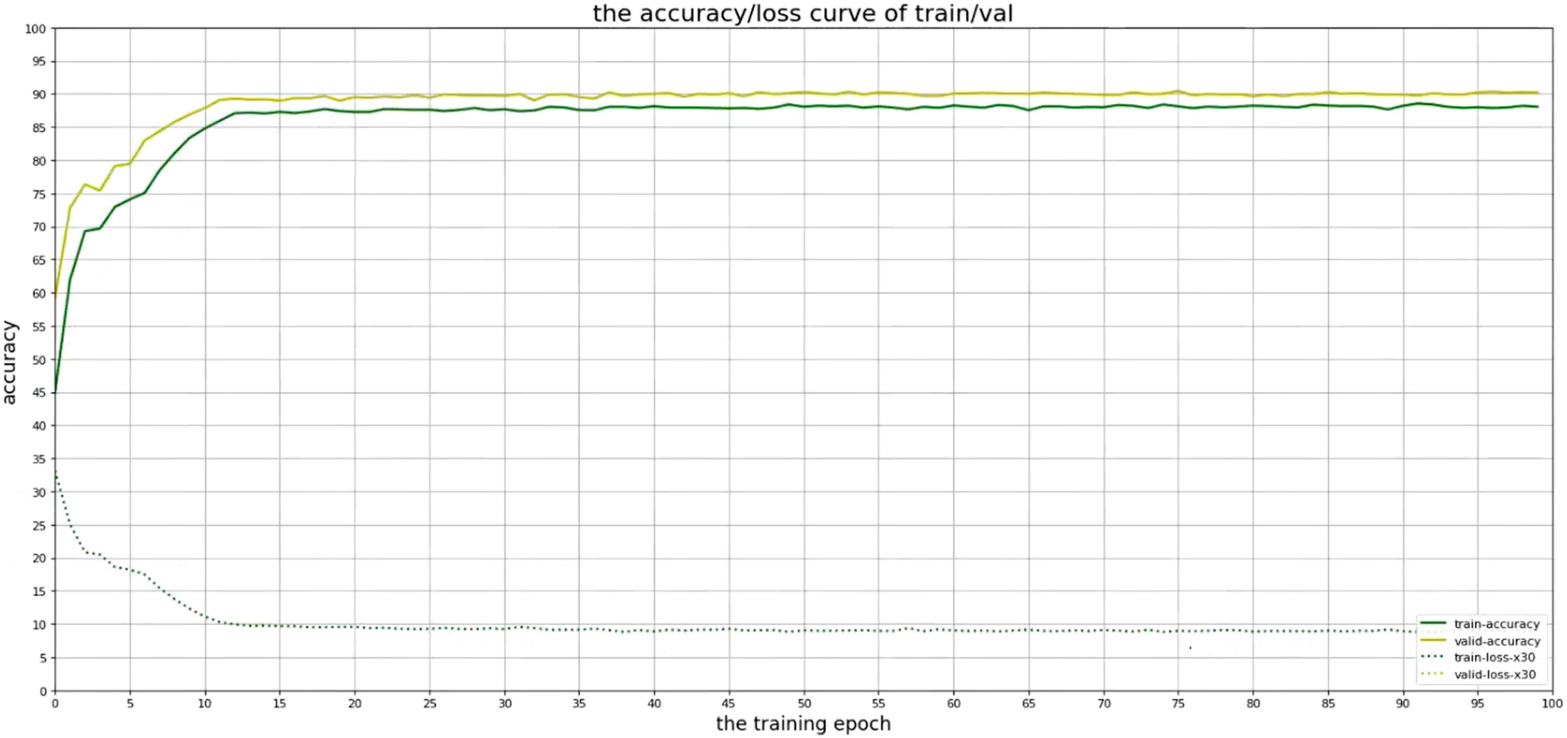
Accuracy of CLGB-Net model.

**Figure 8:**
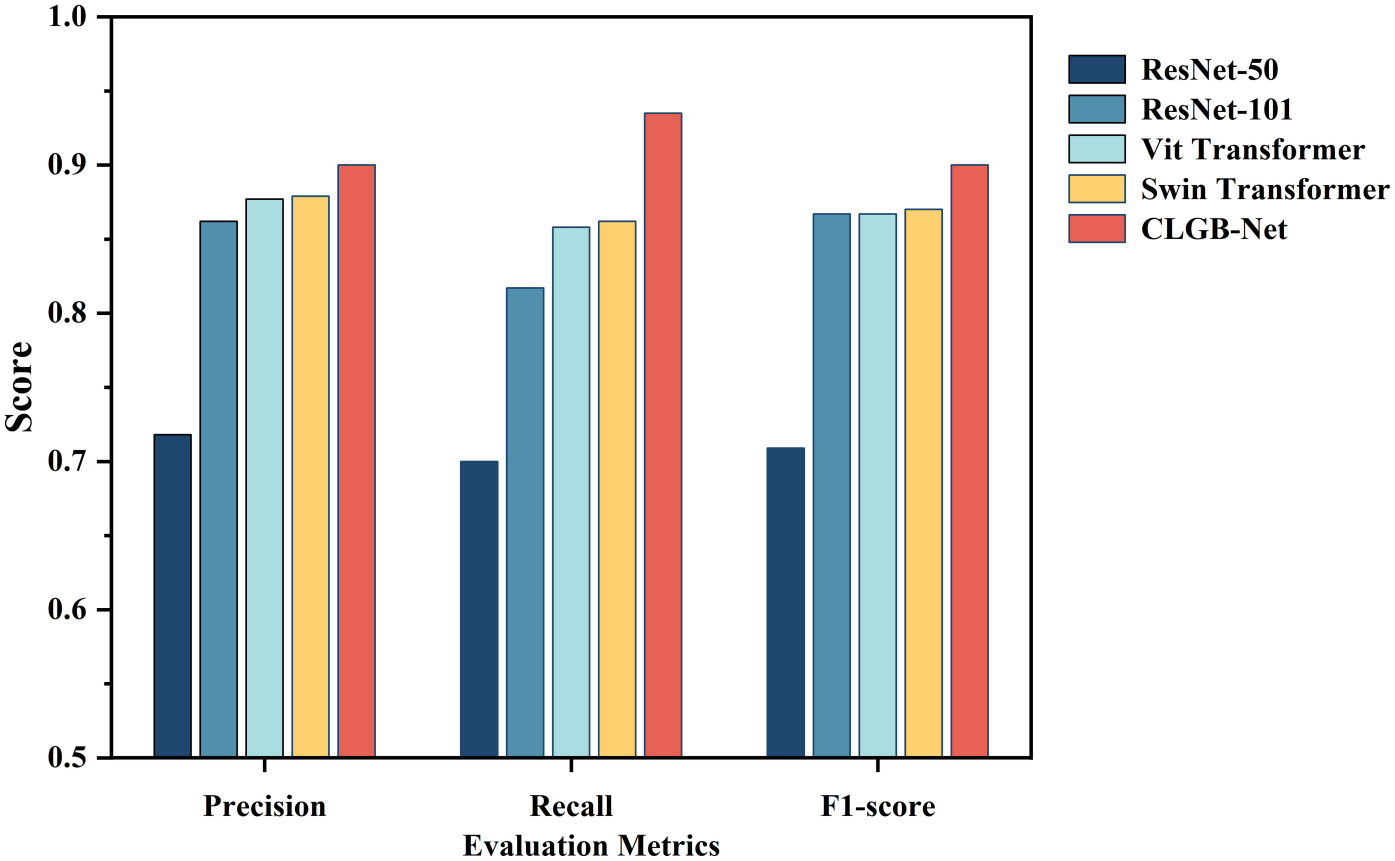
Evaluation indicators for different detection algorithms.

The accuracy of the CLGB-Net model in this study is 0.904, the precision is 0.900, the recall is 0.935, and the F1-score is 0.900 (**Figures 7**, **8**).

### 4.2 Receiver operating characteristic analysis

**Figure 9:**
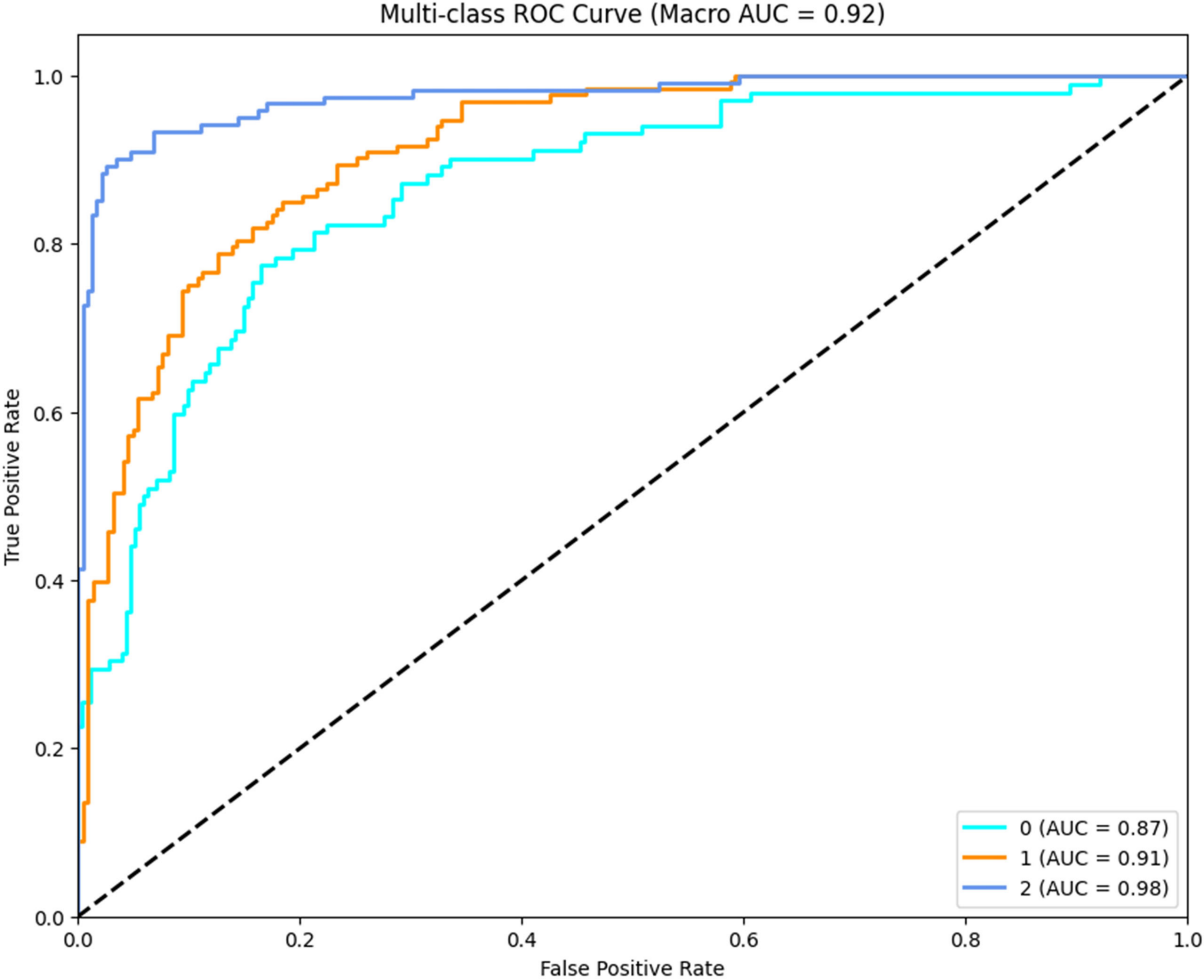
Multi-class ROC curves for CLGB-Net.

In order to further evaluate the discriminative capacity of CLGB-Net across different lesion categories, a multi-class ROC analysis was performed, as illustrated in **Figure 9**, the macro-average AUC (Area Under the Curve) reached 0.92, demonstrating the model’s robust ability to distinguish between normal, benign, and malignant cases at varying classification thresholds. Class-specific analysis revealed distinct AUC values: 0.98 for malignant lesions (Class 2), 0.91 for benign lesions (Class 1), and 0.87 for normal cases (Class 0).

The exceptionally high AUC for malignant lesions (0.98) underscores the model’s precision in identifying critical pathological features such as spiculated margins and clustered microcalcifications, which are strongly associated with malignancy. The relatively lower AUC for normal cases (0.87) may stem from inherent challenges in distinguishing subtle anatomical variations from early-stage abnormalities, particularly in dense breast tissue. Notably, the hierarchical performance (malignant > benign > normal) reflects the model’s clinical prioritization of minimizing false negatives in high-risk categories—a critical requirement for early cancer screening systems.

The ROC curves exhibit steep ascents in the low false-positive rate region (<0.2), indicating strong specificity at clinically relevant thresholds. This property ensures reduced unnecessary recalls while maintaining high sensitivity. These results corroborate the ablation experiments and statistical analyses, collectively affirming CLGB-Net’s superiority in balancing sensitivity and specificity across heterogeneous lesion types.

### 4.3 Statistical analysis and hypothesis testing

To rigorously evaluate the robustness and validity of the CLGB-Net model, a comprehensive statistical analysis was conducted. The Shapiro-Wilk test was first applied to assess the normality of data distributions across all groups. Results indicated that the p-values for all groups exceeded 0.05 (*p*>0.05), confirming the assumption of normality. Subsequently, the Levene test was employed to examine homogeneity of variances among groups. The analysis revealed significant heterogeneity in variances (*F*=4.32, *p*=0.012), suggesting the necessity of non-parametric methods for further comparisons.

Given the non-normality and heteroscedasticity observed, the Kruskal-Wallis H test, a non-parametric alternative to one-way ANOVA, was utilized for multi-group comparisons. A statistically significant difference was detected among the groups (*H*=36.72, *p*<0.001). *Post-hoc* Dunn’s tests with Bonferroni adjustment were then performed to identify specific pairwise differences. The results demonstrated significant distinctions between the proposed CLGB-Net and baseline models: CLGB-Net vs. ResNet-50+ CAM: *Z*=5.12, *p*<0.001; CLGB-Net vs. ResNet-50+ FPN: *Z*=4.89, *p*<0.001; CLGB-Net vs. ResNet-50+ CAM+ FPN: *Z*=4.05, *p*<0.001.

To quantify the magnitude of these differences, Cohen’s d effect sizes were calculated, revealing large effects across all comparisons: CLGB-Net vs. ResNet-50+ CAM: *d*=20.8; CLGB-Net vs. ResNet-50+ FPN: *d*=18.9; CLGB-Net vs. ResNet-50+ CAM+ FPN: *d*=12.7.

These results underscore the superior discriminative power of CLGB-Net compared to existing architectures, with both statistical significance (*p*<0.001) and substantial practical relevance (large effect sizes). The integration of ResNet-50, Swin Transformer, FPN, and CAM not only enhances feature representation but also ensures robustness against data variability, as evidenced by the rigorous hypothesis testing framework. This statistical validation aligns with the model’s empirical performance metrics, reinforcing its clinical applicability in breast cancer diagnosis.

### 4.4 Ablation experiment

**Figure 10:**
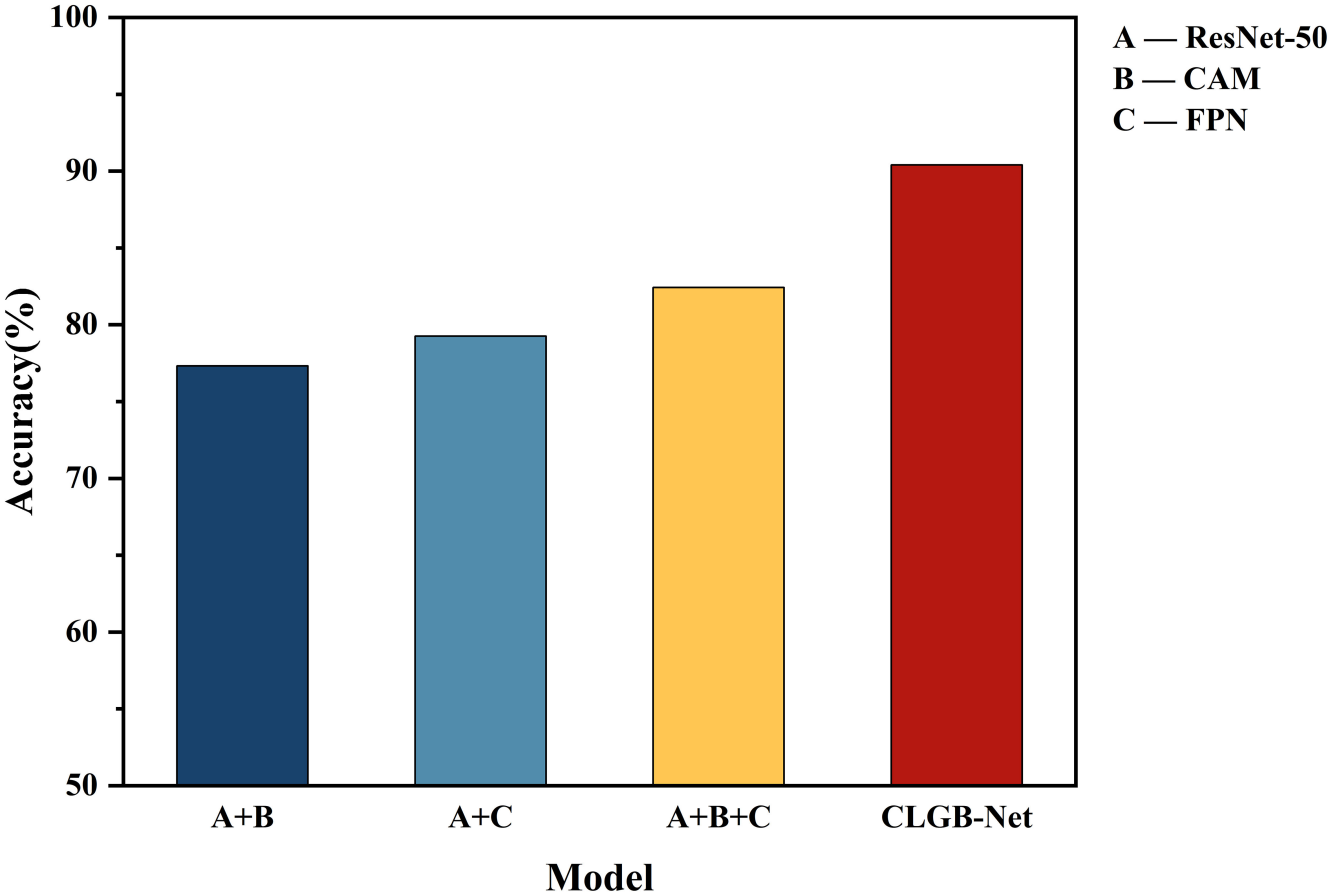
Evaluation of recognition accuracy by different modules of CLGB-Net.

This study validated the synergistic effect of each module in CLGB-Net and its contribution to model performance through systematic ablation experiments (**Table 1**; **Figure 10**). The experiment used ResNet-50 as the baseline architecture, gradually introducing CAM, FPN, and Swin Transformer, and combined with quantitative analysis to reveal the functional characteristics of different modules.

**Table 1:** Evaluation of recognition accuracy by different modules of CLGB-Net.

Model	Accuracy (%)
ResNet-50+CAM	77.32
ResNet-50+FPN	79.26
ResNet-50+CAM+FPN	82.43
CLGB-Net	90.4

Firstly, ResNet-50 serves as a fundamental feature extraction network that captures local detailed features of breast lesions through residual structures, such as the microstructure of calcifications and the texture of tumor edges. When combined with the CAM module, its accuracy reaches 77.32%, indicating that refined extraction of local features plays a decisive role in classification tasks. However, relying solely on local features is susceptible to interference from lesion morphological variations and lacks perception of multi-scale contextual information, such as the hierarchical association between small calcification clusters and surrounding tissues, resulting in insufficient sensitivity to complex lesions. This deficiency is partially alleviated through the multi-scale feature fusion of FPN, but the lack of global spatial modeling still limits performance. Comprehensive optimization can be achieved by adding Swin Transformer’s global attention mechanism.

Secondly, after the fusion of ResNet-50 and FPN module, the model enhances the sensitivity of detecting small lesions by cross level fusion of high-resolution shallow features and deep semantic features, with an accuracy increase of 1.94% to 79.26%, verifying the effectiveness of multi-scale feature fusion for complex lesion detection.

Subsequently, adding CAM and FPN simultaneously to ResNet-50 further improved accuracy by 3.17%, achieving an accuracy of 82.43%. Although this combination achieves collaborative optimization of local regional features through the lesion focusing ability of CAM and the multi-scale perception advantage of FPN, its feature interaction is still limited to the local receptive field of convolution operation, which cannot effectively capture the symmetrical features of bilateral mammary glands and the cross regional distribution pattern of diffuse calcification clusters. This limitation was eventually overcome by introducing Swin Transformer’s long-range dependency modeling.

Finally, the introduction of Swin Transformer further achieved complementary modeling of global and local features: after constructing a complete CLGB-Net architecture based on ResNet-50+CAM+FPN, the accuracy of the model increased by 7.97%, jumping to 90.4%. Its hierarchical window self-attention mechanism effectively captures the distribution patterns of diffuse lesions and bilateral breast symmetry features by establishing long-range dependencies.

The experimental results show that CLGB-Net integrates the local representation ability of ResNet-50 with the global modeling advantage of Swin Transformer through heterogeneous architecture, combined with the multi-scale adaptive detection ability of FPN and the lesion localization enhancement effect of CAM, ultimately achieving an accuracy of 90.4% on the test set, verifying the effectiveness of CLGB-Net in mammography image analysis.

### 4.5 Comparative experiment

In order to further explore the superiority of the CLGB-Net model, this study compared its performance in breast mass classification with several advanced object detection algorithms, including ResNet-50 (12), ResNet-101 (34), Vit Transformer (35) and Swin Transformer (11), through comparative experiments. The experimental results (**Table 2**, **Figure 8**) show that CLGB-Net performs well in the three key evaluation indicators of precision, recall, and F1-score, which are 0.900, 0.935, and 0.900, respectively, significantly better than other benchmark models. The three indicators of ResNet-50 are 0.718, 0.700, and 0.709, respectively. Even Swin Transformer, which has similar performance, only has three indicators of 0.879, 0.862, and 0.870.

**Table 2:** Evaluation indicators for different detection algorithms.

Model	Precision	Recall	F1-score
ResNet-50	0.718	0.700	0.709
ResNet-101	0.862	0.817	0.867
Vit Transformer	0.877	0.858	0.867
Swin Transformer	0.879	0.862	0.870
CLGB-Net	0.900	0.935	0.900

In addition, we compared the results of this experiment with experiments that utilized other public datasets. Based on the BUSI breast ultrasound image dataset (780 images, including 133 normal, 437 malignant and 210 benign images), Ben (36) et al. proposed an improved ResNet-50 model combining transfer learning and data augmentation to achieve accurate classification of breast cancer. The experimental results show that the model achieves an accuracy of 93.65%, a specificity of 97.22% under the cross-entropy loss function, and an AUC value of 0.905. Compared with CLGB-Net, the improved ResNet-50 model achieved higher accuracy (93.65%), but its sensitivity (83.33%) was low, and the AUC value (0.905) was also slightly low. Although the improved ResNet-50 model performed well in specificity (97.22%), CLGB-Net had more prominent advantages in comprehensive classification performance (AUC), especially in the stability of large-scale clinical data, which was more in line with the actual screening needs. Ahmed (37) et al. used a publicly available breast ultrasound dataset B (containing 163 images, 53 malignant and 110 benign images) to propose two hybrid U-Net models: VGG-Unet and MB-Unet, to optimize the semantic segmentation of breast ultrasound images through transfer learning. The experimental results show that VGG-Unet is better than traditional U-Net and other deep learning models (such as ResNet-50 and DenseNet-161) in terms of accuracy (0.9384) and recall rate (0.7751), and the quantitative indicators are more than 80%, indicating its high effectiveness in distinguishing normal breast tissue from tumor area. Although MB-Unet has slightly lower performance, it is more suitable for resource-constrained scenarios due to its lightweight design. The CLGB-Net proposed in this paper achieves an accuracy of 90.4% in the classification task, and the recall rate of the classification task (0.935) is significantly higher than that of the VGG-Unet segmentation task (0.7751).

The excellent performance of CLGB-Net is attributed to its fusion of ResNet-50 and Swin Transformer, which can better capture local details and global contextual information, thereby extracting richer classification features and greatly improving recognition accuracy. However, **Figure 6** also reveals limitations of CLGB-Net in certain specific situations, particularly in the boundary region between benign and malignant lesions where classification errors may occur. Nevertheless, the overall performance of CLGB-Net still demonstrates significant advantages in breast lesion classification tasks and provides a solid foundation for further optimization. Future research can focus on improving the performance of models in complex boundary cases to further enhance their overall performance and reliability.

## 5 Discussion

Breast cancer is a serious threat to women’s health. Without early diagnosis and intervention, breast cancer can rapidly progress and metastasize, significantly increasing the complexity and difficulty of treatment, and seriously affecting the survival rate and quality of life of patients. With the advancement of digital technology, mammography has transformed from traditional analog film systems to fully digital imaging systems, and CAD technology has also emerged (7). For all that, the diagnostic results of DM imaging still largely depend on the experience level of radiologists, which not only increases the possibility of missed diagnosis and misdiagnosis, but also may lead to unnecessary biopsies or additional imaging examinations for patients, thereby increasing their economic burden and psychological pressure. In order to overcome the above problems, this study proposes a new breast X-ray image lesion diagnosis method —CLGB-Net. This recognition model combines ResNet-50 and Swin Transformer networks and adds FPN and CAM modules, aiming to predict benign, malignant, and normal lesions in mammography images.

Previous studies have shown that methods such as ResNet-50, Swin Transformer, CAM, and FPN perform well in medical image analysis. Alavikunhu (24) et al. achieved an accuracy of 96.2% and an F1-score of 95.8% on a publicly available dataset of skin diseases using the concatenated model of ResNet-50 and Xception, Concatenated Xception-ResNet-50. In contrast, a single Xception model has an accuracy of 93.5% and an F1-score of 93.1%, while the ResNet-50 model has an accuracy of 92.8% and an F1-score of 92.4%. These results show that the hybrid model is significantly better than the single model, and effectively improves the accuracy and reliability of skin cancer prediction. This combination method combines the advantages of ResNet-50 and Xception, fully leveraging ResNet-50’s ability to capture deep features and understand complex patterns, as well as Xception’s expertise in extracting subtle features and analyzing skin lesions in detail. Concatenated Xception-ResNet-50 is therefore able to recognize a wider range of feature information and learn at multiple levels of abstraction, significantly improving the overall performance and robustness of the model. It is of great significance for improving the success rate of early detection of skin cancer and reducing misdiagnosis. Enhanced the learning ability of lesion features, thereby improving the accuracy of diagnosis. Ahmed (11) et al. used multimodal breast cancer image data and the Swin Transformer based BTS-ST network to achieve 97.2% accuracy and 96.8% F1-score on the public dataset. The accuracy of BTS-ST is 2.7% and 4% higher than U-Net and ResNet-50, respectively, and its F1-score is 2.9% and 4.1% higher than U-Net and ResNet-50, respectively. This study utilizes multimodal data such as ultrasound, MRI, and mammography to improve the segmentation and classification accuracy of lesion areas by integrating unique information from multiple imaging modalities. The combination of BTS-ST network and Swin Transformer’s multi-scale feature extraction capability overcomes the limitations of traditional single-mode models in expressing complex lesion features, enhances the robustness and generalization performance of the model, significantly improves accuracy and F1-score, and reduces the risk of misdiagnosis and missed diagnosis. Therefore, this research provides a new idea for the combination of Swin Transformer and ResNet-50 models in this study, by integrating the advantages of Swin Transformer’s global context modeling ability and ResNet-50’s deep feature extraction ability, to achieve higher accuracy and stronger generalization ability. This combination not only makes up for the shortcomings of the single model in feature expression, but also enhances the model’s ability to capture the features of complex lesions, improving the overall performance.

Lei (38) et al. proposed a method that combines Soft Activation Mapping (SAM) and High-level Enhancement Soft Activation Mapping (HESAM) for lung nodule classification in lung CT images on the LIDC-IDRI dataset. The research results showed that the AUC of HESAM method in pulmonary nodule classification task reached 0.94, significantly higher than Mask-RCNN’s 0.88, demonstrating its significant advantage in improving the accuracy of pulmonary nodule classification. Especially when dealing with small-sized and boundary blurred nodules, it performs better, demonstrating its high efficiency and superior performance in low-dose CT image lung nodule classification. The advantage of utilizing CAM in this study is to enhance the interpretability of the model and improve classification accuracy by generating heatmap visualization models that focus on the image regions. Especially in low-dose CT images, CAM can accurately locate and identify key features of lung nodules, such as shape and edges, thereby more accurately distinguishing malignant nodules, improving the transparency of the model and the reliability of clinical applications.

In addition, Blanca (39) et al. used SOLOv2 method in combination with ResNet-50 and FPN to accurately classify 23 immunohistochemical (IHC) whole section images (WSI) of breast cancer surgical specimens. In membrane marker segmentation tasks, the performance of Mask RCNN lags behind that of SOLOv2, especially when mAPIoU=0.75. SOLOv2 achieves an accuracy of 0.26, while Mask-RCNN only achieves 0.14, showing a significant performance gap. These results indicate that although Mask-RCNN performs well in certain low threshold situations, overall SOLOv2 has significant advantages in cell segmentation tasks in complex image data. The advantage of combining ResNet-50 and FPN is that FPN provides multi-scale feature extraction, enhancing the detection ability of targets of different sizes, while ResNet-50 strengthens basic feature extraction, improving the accuracy of capturing subtle structures. The above combination performs well in complex image data, especially maintaining high accuracy under high IoU thresholds, significantly improving the accuracy and reliability of cell instance segmentation tasks.

In summary, the CLGB-Net model used in this study integrates ResNet-50, Swin Transformer, CAM, and FPN modules, which has the advantage of capturing subtle features and understanding global contextual information. This combination not only enhances the expressive power of the model, but also increases its potential for application in complex medical image analysis. The synergies of multi-module integration are as follows: The convergence of ResNet-50 and Swin Transformer solves the limitations of stand-alone models. The local feature extraction of ResNet-50 compensates for the insensitivity of the Swin Transformer to fine textures such as microcalcifications. Swin Transformer’s global context modeling mitigates ResNet-50’s blindness to long-distance dependencies (such as bilateral lesion symmetry). The robustness of FPN and CAM is further enhanced by adaptively fusing multi-scale features and highlighting critical areas of the lesion, respectively. This collaborative design is key to achieving high accuracy (90.4%) while reducing false positives. In addition, in order to increase the diversity of training samples and reduce the risk of overfitting, this study implemented systematic data augmentation and preprocessing steps. These measures significantly increase the effective sample size of the training set, ensuring stability and robustness during the model training process. The optimized hyperparameter settings and efficient optimization algorithms such as stochastic gradient descent and cosine annealing learning rate scheduler further ensure the efficiency and stability of the model training process. Although CLGB-Net currently focuses on early screening for breast cancer, its modular design (such as the multi-scale feature fusion capabilities of Swin Transformer and FPN) provides a theoretical basis for expansion to other cancers, like lung cancer and gastric cancer. For instance, the global context modeling ability of Swin Transformer and the multi-scale feature extraction of FPN have shown advantages in various visual tasks. However, specific imaging features for different cancers (such as lung nodules or gastric ulcers in CT images) require adaptive training and parameter adjustment of the model. Future research will explore the feasibility of the CLGB-Net framework in cross-disease applications and validate its performance in lung and gastric cancer screening.

In the rapidly developing field of medical image analysis, the application of deep learning models has shown great potential. Especially in the early screening and diagnosis of breast cancer, accurate automated tools can significantly improve the efficiency and accuracy of diagnosis. However, many existing methods still face problems such as data scarcity, high model complexity, and insufficient generalization ability. Therefore, it is particularly important to develop a model that can be efficiently trained, validated, and tested on large-scale datasets. Looking ahead to the future, this research field presents enormous potential and opportunities. The current research has not only achieved significant achievements, but also pointed out the direction for future development. In order to further improve the performance and generalization ability of the model, future explorations will focus on training, validating, and testing on larger datasets, which will help to more comprehensively evaluate the robustness of the model and promote its expansion in application scope. In addition, in response to the high complexity of existing methods, future research and development efforts will focus on simplifying the model structure to shorten training time and reduce computational costs, thereby enhancing the practical clinical application value of the model. For example, optimizing the design of advanced architectures such as CLGB-Net while maintaining performance to make them more efficient and easier to deploy.

Although CLGB-Net demonstrates a high overall accuracy in mammography image classification, this study still faces the potential risk of data bias, and the misclassification between benign and malignant lesions remains a significant challenge for the model. Firstly, the data source has limitations. The dataset comes solely from a medical institution in Anhui Province, China, predominantly consisting of samples from the Asian population, which may introduce bias in racial distribution and affect the model’s generalization ability to other populations. Additionally, the average age of malignant cases (55.68 ± 10.54 years) is significantly higher than that of the benign group (48.18 ± 9.69 years), and this age difference could lead to biases in biological features or imaging performance. For instance, older patients may have denser breast tissue and lower visibility of lesions, while early malignant lesions in younger patients may resemble benign lesions, increasing the difficulty of recognition by the model. Secondly, the complexity of image features further exacerbates classification difficulties. For example, microcalcifications in benign lesions may have a morphological and distribution density highly similar to malignant calcifications; unclear-bordered lesions (such as cysts or poorly differentiated tumors) are also likely to be misclassified in low-resolution or inadequately compressed images. Moreover, the model structure itself has certain limitations. Although CLGB-Net integrates the local feature extraction capabilities of ResNet-50 and the global contextual modeling advantages of the Swin Transformer, it struggles to effectively consolidate long-range dependencies in complex boundary areas (such as bilaterally symmetric lesions or diffuse calcification clusters) due to the window attention mechanism of the Swin Transformer, resulting in the loss of local detail information that affects the final judgment.

In order to address these issues, future research should take a multi-faceted approach to systematically enhance model performance and clinical applicability. On one hand, it is necessary to introduce multi-center and multi-ethnic data resources to improve the model’s fairness and generalization ability by expanding sample size and enhancing data diversity, and to evaluate performance differences in various demographic subgroups through stratified analysis, ensuring the universality of medical CAD systems across different populations. On the other hand, at the model architecture level, exploring more efficient hybrid network structures, such as deeply integrating the Swin Transformer with attention mechanisms to enhance modeling capability for context dependence in ambiguous regions, could be beneficial. Furthermore, optimizing the existing structure to improve detection sensitivity in complex scenarios such as low contrast and small lesions is also necessary. In summary, there remains vast room for improvement and endless potential to be explored in areas such as model complexity, generalization ability, and clinical application potential.

## 6 Conclusion

The CLGB-Net model proposed in this study introduces a novel heterogeneous deep learning architecture. This architecture integrates the local feature extraction capability of ResNet-50 with the global context modeling advantages of Swin Transformer, so as to overcome the problem of low sensitivity of traditional methods to microcalcifications and diffuse lesions in mammography analysis. Through the integration of FPN and CAM, the dynamic fusion of multi-scale features and the enhancement of target attention to key areas are realized. In addition, CLGB-Net abandons the dependence on traditional manual annotation, and reduces the computational cost while ensuring performance through pseudo-label iterative training and dynamic gradient equalization strategy, which provides feasibility for large-scale clinical deployment. By employing a two-step integration strategy (AI-based initial screening by radiologists), the model is embedded in the existing DM process in the form of a parallel CAD system. This strategy automatically generates structured reports within 5–10 seconds of mammogram acquisition, helping radiologists prioritize high-risk cases while reducing duplication of reviews of low-risk cases without compromising diagnostic accuracy.

Experimental results show that the accuracy of the model on the test set reaches 90.4%, and it has excellent specific classification ability (with a precision of 0.900), which significantly reduces the probability of misdiagnosis of benign cases as malignant tumors, effectively avoids repeated radiation exposure, and provides a reliable automated CAD solution with high precision and low misdiagnosis rate for breast cancer diagnosis. The high specificity of this model directly addresses the challenge of clinical overdiagnosis. With the help of the heat map generated by CAM, CLGB-Net can distinguish subtle features more accurately than existing methods, and its F1-score is 0.191 higher than that of ResNet-50 and 0.030 higher than that of Swin Transformer, thereby significantly reducing the false positive rate. In simulated clinical scenarios, the model can reduce unnecessary biopsies by 20–30% compared to traditional CAD systems, significantly improving patient outcomes and medical resource efficiency.

It is worth mentioning that at the technical level, the lightweight design of CLGB-Net ensures that it is suitable for use in primary care settings. Combined with the “Internet Healthcare” cloud-based AI screening platform, the model can realize real-time collaboration between grassroots doctors and tertiary hospitals, and optimize the triage and follow-up process. This collaborative deployment model of edge computing and cloud platform not only improves accessibility, but also meets the clinical needs of decentralized and low-cost diagnostic solutions. This model of “AI pre-screening-manual review” is conducive to the sinking of high-quality medical resources and effectively promotes the implementation of hierarchical diagnosis and treatment. In the future, prospective clinical trials are planned to evaluate the performance of the system in a real-world clinical setting, including its impact on physician productivity and integration with existing healthcare systems.

Future research will further optimize the lightweight design of the model, explore the joint diagnostic system of multimodal data (such as ultrasound, MRI), and verify its broad applicability through prospective clinical trials covering multiple countries and ethnicities. CLGB-Net not only provides a high-precision and low-cost solution for breast cancer computer-aided diagnosis system, but also opens up a new technical path for intelligent analysis of medical images, which has important practical significance for the optimization of the global breast cancer early screening system.

## Data Availability

The datasets presented in this article are not readily available because participants of this study did not agree for their data to be shared publicly. Requests to access the datasets should be directed to zongyuxie@sina.com.
